# Is there any relation between Duration of breastfeeding and anemia?

**Published:** 2015-12-10

**Authors:** H Dalili, A Baghersalimi, S Dalili, F Pakdaman, A Hassanzadeh Rad, M Abbasi Kakroodi, SM Rezvany, Sh Koohmanaei

**Affiliations:** 1 Department of Pediatrics, Breastfeeding Research Center, Imam Khomeini Hospital, Tehran University of Medical Sciences, Tehran, Iran.; 2 Pediatrics Growth Disorders Research Center, School of Medicine, 17th Shahrivar Hospital, Guilan University of Medical Sciences, Rasht, Iran.; 3 Guilan Health Center, Guilan University of Medical Sciences, Guilan, Iran.

**Keywords:** Anemia, Breast Feeding, Ferritin, Iron-Deficiency

## Abstract

**Background:**

In the early months of life, Breastfeeding increases chance of survival, reduces recovery time after disease and mortality due to infections such as diarrhea and acute respiratory infections. However, infants who are exclusively breast-fed for more than 6 months in developing countries may be at increased risk of anemia. Therefore, the aim of study was to assess the relation between duration of breastfeeding and anemia.

**Materials and Methods:**

In this analytical cross-sectional study, 400 neonates registered in primary health care system since birth time. Complete blood count and serum ferritin were obtained. Data were analyzed by chi- square test and regression analysis. P-value less than 0.05 was considered significant and 95% confidence interval was noted.

**Results:**

Results of this study showed that 199 infants were anemic (Hemoglobin (Hb) concentration <11 mg/dl). Ten percent of anemic patients reported Ferritin< 12ng/dl and %25 of anemic children had iron deficiency anemia (IDA). In Binominal logistic regression, merely kind of delivery and duration of breastfeeding were effective factors. Binominal logistic regression also showed that natural vaginal delivery and exclusive breastfeeding up to 6 months had a significant influence on anemia. Exclusive breast feeding for 6 months or more increased the likelihood of anemia. In addition, 4 months exclusive breastfeeding decreased 0.686 fold the likelihood of anemia.

**Conclusion:**

According to the results, it seems that revision of health program recommendations for iron supplementation can be constructive. National planning to promote the level of knowledge regarding natural vaginal delivery and appropriate period for clamping can be recommended.

## Introduction

In the early months of life, breastfeeding increases chance of survival, reduces recovery time after disease and mortality due to infections such as diarrhea and acute respiratory infections (1) According to previous investigations, exclusive breast-feeding(EBF) efficiently decreased rates of various diseases such as infections, obesity, atherosclerosis, hypertension, rheumatic diseases, gastrointestinal disorders, nutritional deficiencies, asthma, and diabetes (2-4). Therefore, in 2001, world health organization (WHO) recommended exclusive breast-feeding (EBF) for the first 6 months of life. (5) also, in 2011, Cochrane review recommended it for both developing and developed countries(6).

However, Infants who are exclusively breast-fed for more than 6 months in developing countries may be at increased risk of anemia(7). The numerous causes of anemia include iron deficiency (about 50% of all cases), nutritional deficiencies such as vitamins B12, B6, A, riboflavin, and folic acid; chronic diseases and inflammation. These conditions cause blood loss or hemolysis and hemoglobinopathies. 

Iron deficiency is the most common and widespread nutritional disorder, occurring both in developed and developing countries.(8) Although, breast milk has a relatively small amount of iron, its absorption is high(9). 

Therefore, based on previous lines of evidence, exclusive breastfeeding protects children from Iron deficiency anemia in the first 4 months of life. 

After this period, the findings of previous studies in agreement with the literature, demonstrated an increase in anemia and iron deficiency rates. (10-12) After 6 months, breast milk cannot provide all iron requirements as a result of depletion of stored iron, increased body size and total red blood cell count. (13-15). The aim of this study was to assess whether there is any relation between duration of breastfeeding and anemia.

## Materials and Methods

In this cross sectional analytic study, four hundred neonates registered in primary health care system since birth time. Eligible participants were selected by cluster sampling from 16 urban and rural health care centers in Rasht, Guilan province, Iran. Inclusion criteria were those ranged between 6 to 9 months, birth weight between 2500-4000 g with term delivery. Exclusion criteria were considered as history of blood transfusion, failure to thrive, gross physical anomaly, genetic or chromosomal disorders, and any chronic diseases or febrile diseases during or 2 weeks preceding laboratory evaluation. 

 Data were gathered by a checklist which consisted of demographic characteristics such as age, sex, gravida, parity, place of inhabitants (rural /urban), feeding status (breastfeeding, mix, and formula), maternal age and history of anemia. CBC indices (WBC, RBC, Hb, and HCT) and serum ferritin were calculated to assess iron deficiency anemia. Anemia was defined by World Health Organization (WHO) as Hb concentration <11m g/dl. The severity of anemia is defined as mild (Hemoglobin: Hb: 10-10.9 g/dl), moderate (7-9.9 g/dl) and severe (<7g/dl) (16).Based on cutoffs from National Health and Nutrition Examination Surveys[NHANES II], (17, 18) NHANES III,(19, 20)] and CDC publications,(18, 20), HB < 110 g/L and at least 2 abnormalities with MCV < 74 fl and RDW > 14 also indicated as iron deficiency anemia (IDA). Ethical approval was obtained from Tehran University of Medical Sciences (26163-10-10-92) and consent letter was taken from parents. Statistical analysis was performed using SPSS software (version 16, SPSS Inc, Chicago, IL, USA). Data were reported by descriptive statistics (frequency, percent) and analyzed by chi- square and regression analysis P-value less than 0.05 considered significant and 95% confidence interval was noted. 

## Results

Four hundred infants aged between 6to 9 months were entered to the study. Results showed that 199 infants (49.5%) were anemic (Hb <11 g/dl). According to the results, 131( 65.8% ) and 68(34.2% ) patients suffered from mild and moderate anemia, respectively. In moderate anemic patients, 56 patients (82.4%) were reported with hb range from 9-9.9. Based on Ferritin< 12mg/dl and NHANES III criteria, the results showed that out of all anemic patients, 20 (10%) and 50 (25% ) had iron deficiency anemia, respectively.

Patients characteristics based on feeding status were summarized in Table I. This table showed that there was significant difference in feeding status (p<0.05) in terms of maternal education and occupation, and paternal education.

 In multinomial regression only maternal job was an effective factor. Employed mothers preferred mix feeding in comparison with breastfeeding 0.221 (0.75 - 0.650). 

There was no significant difference between anemic and non anemic infants regarding place of inhabitants, parity, gravid, and sex (p>0.05) (Table 2).

Odds ratio of kind of delivery , maternal job, and graduated mother were 0.44 ( 95%CI: 0.258-0.749), 0.42 (95% CI: 0.189-0.934), and 1.792( 95%CI: 1.062-3.025), respectively . The results showed significant statistical difference between anemic and non anemic infants regarding nutritional type and duration of breast feeding (P=0.004, 0.0001, respectively)

 However, in Binominal logistic regression merely kind of delivery and duration of breast feeding were effective. Binominal logistic regression showed that natural vaginal delivery and 6 months exclusive breastfeeding had significant influence on anemia (p<0.05).

Increased exclusive breast feeding for about 6 months increased the likelihood of anemia. In addition, decreasing exclusive breastfeeding for about 4 months increased 0.314 fold the absence of anemia (- 68.6%) (Table III).

**Figure 1:**
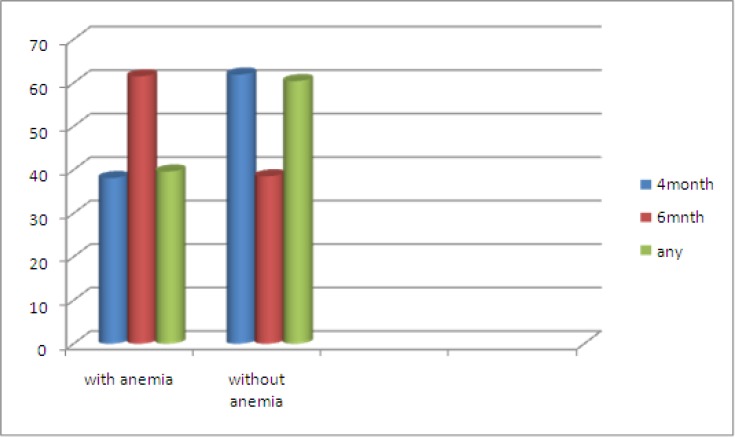
Duration of breastfeeding in IDA and non IDA groups

Prevalence of anemia in 6months breast fed infants was high in comparison with other groups (P=0.000) and there was no significant difference in the prevalence of anemia between 4months breast feeding and formula fed infants (p>0.05) (Figure1).

**Table I:** Patients characteristics based on feeding status

			**nutritional state**				
			**bf**	**Formula**	**mix**	**Total**	**P value**
**Place of inhabitants**	**urban**	**Count**	**112**_a_	**11**_a_	**18**_a_	**141**	**.179**
		**Percent** ^a^	**61.9**	**61.1**	**81.8**	**63.8**	
	**rural**	**Count**	**89**_a_	**13**_a_	**6**_a_	**108**	
		**Percent** ^a^	**38.1**	**38.9**	**18.2**	**36.2**	
** Type of delivery**	**CS**	**Count**	**131**_a_	**16**_a_	**19**_a_	**166**	**.121**
		**Percent** ^a^	**72**	**88.9**	**86.4**	**74.8**	
	**NVD**	**Count**	**51**_a_	**2**_a_	**3**_a_	**56**	
		**Percent** ^a^	**28**	**11.1**	**13.6**	**25.2**	
**maternal education **	**≤diploma**	**Count**	**163**_a_	**13**_b_	**16**_b_	**253**	**.017**
		**Percent** ^a^	**89.6**	**72.2**	**72.7**	**86.5**	
	**>diploma**	**Count**	**19**_a_	**5**_b_	**6**_b_	**30**	
		**Percent** ^a^	**10.4**	**27.8**	**27.3**	**13.5**	
**maternal job**	**employed**	**Count**	**5a**	**1a,b**	**4b**	**10**	**.004**
		**Percent** ^a^	**2.7**	**5.6**	**18.2**	**4.5**	
	**unemployed**	**Count**	**177**_a_	**17**_a_ **,**_b_	**18**_b_	**212**	
		**Percent** ^a^	**97.3**	**94.4**	**81.8**	**95.5**	
**Paternal education**	**≤diploma**	**Count**	**168**_a_	**13**_b_	**18**_a,b_	**262**	**.013**
		**Percent** ^a^	**92.3**	**72.2**	**81.8**	**89.6**	
	**>diploma**	**Count**	**14**_a_	**5**_b_	**4**_a,b_	**23**	
		**Percent** ^a^	**7.7**	**27.8**	**18.2**	**10.4**	
**parity**	**1**	**Count**	**105**_a_	**14**_a_	**15**_a_	**134**	**.351**
		**Percent** ^a^	**59.7**	**77.8**	**71.4**	**62.3**	
	**2**	**Count**	**60**_a_	**4**_a_	**6**_a_	**70**	
		**Percent** ^a^	**34.1**	**22.2**	**28.6**	**32.6**	
	**3**	**Count**	**11**_a_	**0**_a_	**0**_a_	**15**	
		**Percent** ^a^	**6.3**	**.0**	**.0**	**5.1**	
**gravida**	**1**	**Count**	**99**_a_	**13**_a_	**13**_a_	**125**	**.718**
		**Percent** ^a^	**55**	**72.7**	**59.1**	**56.8**	
	**2**	**Count**	**63**_a_	**5**_a_	**9**_a_	**77**	
		**Percent** ^a^	**35**	**27.8**	**40.9**	**35**	
	**3**	**Count**	**15**_a_	**0**_a_	**0**_a_	**15**	
		**Percent** ^a^	**8.3**	**0**	**0**	**6.8**	
	**4**	**Count**	**2**_a_	**0**_a_	**0**_a_	**2**	
		**Percent** ^a^	**1.1**	**0**	**0**	**.9**	
**sex**	**male**	**Count**	**100**_a_	**10**_a_	**14**_a_	**124**	**.74**
		**Percent** ^a^	**54.9**	**55.6**	**63.6**	**55.9**	
	**female**	**Count**	**82**_a_	**8**_a_	**8**_a_	**98**	
		**Percent** ^a^	**45.1**	**44.4**	**36.4**	**44.1**	

**Table II:** demographic characteristics in anemic and non anemic infants

			**Anemia in infant**			
			**yes**	**no**	**Total**	**P value**
***area location***	***urban***	***Count***	**125**_a_	**142**_a_	**267**	**.162**
		***Percent*** ^a^	**63.1**	**69.6**	**66.4**	
	***rural***	***Count***	**73**_a_	**62**_a_	**135**	
		***Percent*** ^a^	**36.9**	**30.4**	**33.6**	
***delivery***	***CS***	***Count***	**148**_a_	**175**_b_	**323**	**.012**
		***Percent*** ^a^	**74.4**	**85.8**	**80.1**	
	***NVD***	***Count***	**50**_a_	**29**_b_	**79**	
		***Percent*** ^a^	**25.1**	**14.2**	**19.6**	
***Mom job***	***employed***	***Count***	**7**_a_	**21**_b_	**28**	**.007**
		***Percent*** ^a^	**3.5**	**10.3**	**6.9**	
	***unemployed***	***Count***	**192**_a_	**183**_b_	**375**	
		***Percent*** ^a^	**96.5**	**89.7**	**93.1**	
***Mom education ***	***Under graduated***	***Count***	**174**_a_	**159**_b_	**343**	**.012**
		***Percent*** ^a^	**87.4**	**77.9**	**82.6**	
	***graduated***	***Count***	**25**_a_	**49**_b_	**70**	
		***Percent*** ^a^	**12.6**	**22.1**	**17.4**	
***Dad education***	***Under graduated***	***Count***	**181**_a_	**170**_a_	**351**	**.023**
		***Percent*** ^a^	**91**	**83.3**	**87.1**	
	***graduated***	***Count***	**18**_a_	**34**_a_	**52**	
		***Percent*** ^a^	**9**	**16.7**	**12.9**	
***number delivery***	***1***	***Count***	**119**_a_	**129**_a_	**248**	**.544**
		***Percent*** ^a^	**61.3**	**65.1**	**63.2**	
	***2***	***Count***	**64**_ a_	**61**_a_	**125**	
		***Percent*** ^a^	**33**	**31.3**	**32.1**	
	***3***	***Count***	**11**_a_	**7**_a_	**18**	
		***Percent*** ^a^	**5.7**	**3.6**	**4.6**	
***Number gravid***	***1***	***Count***	**110**_a_	**120**_a_	**230**	**.568**
		***Percent*** ^a^	**55.8**	**59.4**	**57.6**	
	***2***	***Count***	**69**_a_	**60**_a_	**129**	
		***Percent*** ^a^	**35**	**29.7**	**32.3**	
	***3***	***Count***	**15**_a_	**18**_a_	**33**	
		***Percent*** ^a^	**7.6**	**8.9**	**8.3**	
	***4***	***Count***	**2**_a_	**4**_a_	**6**	
		***Percent*** ^a^	**1**	**2**	**1.5**	
***SEX***	***male***	***Count***	**109**_a_	**120**_a_	**229**	**.447**
		***Percent*** ^a^	**54.8**	**58.8**	**56.8**	
	***female***	***Count***	**89**_a_	**84**_a_	**173**	
		***Percent*** ^a^	**44.7**	**41.2**	**42.9%**	
		***Percent*** ^a^	**26.9**	**16**	**21.4**	
***Nutritional state***	***bf***	***Count***	**170**_a_	**132**_b_	**302**	**.00001**
		***Percent*** ^a^	**85.4**	**68.2**	**75.7**	
	***formula***	***Count***	**11**_a_	**38**_b_	**49**	
		***Percent*** ^a^	**5.5**	**19**	**12.3**	
	***mix***	***Count***	**18**_a_	**30**_a_	**48**	
		***Percent*** ^a^	**9**	**15**	**12**	
***Duration of breast feeding***	***4m***	***Count***	**8**_a_	**13**_a_	**21**	**0.000**
		***Percent*** ^a^	**4.1**	**6.5**	**5.3**	
	***6***	***Count***	** 174**_a_	**123**_b_	**282**	
		***Percent*** ^a^	**83**	**61.2**	**71.4**	
	***any***	***Count***	**26**_a_	**65**_b_	**91**	
		***Percent*** ^a^	**13.4**	**32.3**	**23**	

**Table III:** The effect of NVD and 6 months exclusive breastfeeding on anemia by Binominal logistic regression

		B	S.E.	Wald	df	Sig.	Exp(B)
Step 1^a^	exclubf			21.314	2	.000	
	4months	-.423	.508	0.693	1	.405	.655
	6months	-1.192	.266	20.042	1	.000	.304
	Constant	.908	.237	14.697	1	.000	2.480
Step 2^b^	Delivery NVD^b^	.585	.271	4.638	1	.031	1.794
	exclubf			19.863	2	.000	
	4months	-.424	.511	.690	1	.406	.654
	6months	-1.159	.268	18.742	1	.000	.314
	Constant	.412	.329	1.569	1	.210	1.510

a . Variable(s) entered on step 1: exclubf meant exclusive breast feeding

b . Variable(s) entered on step 2: delivery.NVD meant natural vaginal delivery.

## Discussion

Discussion: In this study, it was found that about half of the participants were anemic, 10 and 25 percent of them had iron deficiency anemia based on ferritin and NHANES III criteria, respectively. However, in the third world countries (21), 40 percent of 9 months infants were anemic. WHO publications estimated that prevalence of anemia in south American infants aged between 6 to 9 months 70 percent (22) . The result of this study revealed much higher prevalence in developed countries. They reported 16.2% of infants aged between 6to11 months had Hb< 110 g/L(23); therefore, it seems that anemia in Iranian infants is a severe public health problem and need effective intervention to tackle it. According to WHO (16), more than 40% of anemia was mentioned in severe category of public health. According to previous investigation, it is estimated that the prevalence of iron deficiency anemia is about half of total anemia prevalence(24). Investigators noted nutritional deficiencies (such as vitamins B12, B6, A, riboflavin, and folic acid). Chronic diseases and inflammations are conditions that cause blood loss or hemolysis and hemoglobinopathies. These are remaining causes of anemia in infants (22). Based on this estimation, one can assume that about one hundred of the participants should have iron deficiency anemia, but results of recent study showed that 50 infants had IDA based on NHANES III criteria and only 20 infants had IDA based on serum ferritin<12 ng/dl. Ferritin is an acute phase reactant and common inflammatory diseases such as infections can increase it. American Academy of Pediatrics defines iron deficiency as serum ferritin<30 ng/dl during inflammation (25). Unfortunately researchers could not check CRP, so perhaps some of the anemic infants with serum ferritin> 12ng/dl might have a kind of common infections. Even with more deliberated NHANES III criteria, 25 percent of anemic infants (50 infants) had Iron deficiency Anemia. Therefore, further investigations focusing on etiologies can be recommended. On the other hand, regarding the importance of iron deficiency on growth and development, further investigations indicating transferrin receptor, zinc protoporphyrin, CRP along with frerritin may be more useful to address exact prevalence of IDA. Seventy five percent (149 infants) of anemic participants were not iron deficient (based on NHANESIII). This may be due to unrecognized cases of endemic RBC disorders in the north of Iran such as beta- thalassemia minor, G-6PD deficiency, spherocytosis, sickle cell anemia and its related disorders (eg. Sickle- cell anemia or even α thalassemia. In this study anemic infants had significantly longer exclusive breast feeding than other infants (duration: 4months vs. 6moths). Unfortunately, based on unexpected low number of iron deficient infants compared to total anemics, investigators could not detect significant relation between iron deficiency and the duration of exclusively breast feeding.(26-28). So, revision of current recommendations about the time to start Iron Supplements can be suggested. The result of this study showed higher prevalence of the anemia in infants who born by NVD. Therefore, it seems that early clamping may be noted as an important issue and longitudinal studies can be suggested. McDonald et al. and Hutton et al. mentioned significant association between immediate clamping and anemia. They noted that clamping of the umbilical cord for at least 2 minutes after birth is a favorable method (29,30). The result of this study also showed no significant relation between sex and anemia which was consistent with previous Iranian investigations by Kadivar et al. (31) and Karimi et al.(32). They noted no statistically significant relation between iron-deficiency anemia and sex. However, Domellöf et al. found substantial sex differences in hemoglobin concentration and other hematologic iron indices during infancy (33). Since employed mothers were busy and did not have sufficient time, they preferred mix feeding in comparison with non-employed ones. 

## Conclusion

According to high prevalence of anemia in infants from 6 to 9 months of age in Guilan province, northern Iran, anemia is a severe public health problem. This study showed that duration of exclusively breast feeding, type of delivery, and maternal occupation were significantly related to anemia in this age group. More detailed studies for precise estimation of iron deficiency as well as other nutritional deficiencies along with endemic RBC disorders are needed. It also seems that revision of health program recommendations for iron supplementation can be constructive.
